# Assessment and Treatment for Coronary Microvascular Dysfunction by Contrast Enhanced Ultrasound

**DOI:** 10.3389/fcvm.2022.899099

**Published:** 2022-06-20

**Authors:** Junzhen Zhan, Longhe Zhong, Juefei Wu

**Affiliations:** Department of Cardiology, Nanfang Hospital, Southern Medical University, Guangzhou, China

**Keywords:** coronary microvascular dysfunction (CMD), myocardial contrast echocardiography (MCE), contrast enhanced ultrasound (CEUS), coronary flow, myocardial ischemia

## Abstract

With growing evidence in clinical practice, the understanding of coronary syndromes has gradually evolved out of focusing on the well-established link between stenosis of epicardial coronary artery and myocardial ischemia to the structural and functional abnormalities at the level of coronary microcirculation, known as coronary microvascular dysfunction (CMD). CMD encompasses several pathophysiological mechanisms of coronary microcirculation and is considered as an important cause of myocardial ischemia in patients with angina symptoms without obstructive coronary artery disease (CAD). As a result of growing knowledge of the understanding of CMD assessed by multiple non-invasive modalities, CMD has also been found to be involved in other cardiovascular diseases, including primary cardiomyopathies as well as heart failure with preserved ejection fraction (HFpEF). In the past 2 decades, almost all the imaging modalities have been used to non-invasively quantify myocardial blood flow (MBF) and promote a better understanding of CMD. Myocardial contrast echocardiography (MCE) is a breakthrough as a non-invasive technique, which enables assessment of myocardial perfusion and quantification of MBF, exhibiting promising diagnostic performances that were comparable to other non-invasive techniques. With unique advantages over other non-invasive techniques, MCE has gradually developed into a novel modality for assessment of the coronary microvasculature, which may provide novel insights into the pathophysiological role of CMD in different clinical conditions. Moreover, the sonothrombolysis and the application of artificial intelligence (AI) will offer the opportunity to extend the use of contrast ultrasound theragnostics.

## Introduction

It has been gradually recognized that coronary microvascular dysfunction (CMD) is emerging as a major cause of myocardial ischemia in patients with angina symptoms but in the absence of obstructive coronary artery disease (CAD) as well as in other clinical conditions, including obstructive CAD, primary cardiomyopathies, heart failure with preserved ejection fraction (HFpEF) (1, 2). CMD refers to the spectrum of structural and functional abnormalities in coronary microcirculation, resulting in a blunted coronary blood flow (CBF) and coronary flow reserve (CFR), which can be detected by several invasive or non-invasive methods (3). With the advent and development of current imaging modalities, there is growing evidence in diagnosing and treating patients involved with CMD, making it possible to advance the understanding of the role of CMD across different cardiovascular diseases (4–6).

Despite currently positron emission tomography (PET) is the most used tool for quantitative assessment of the coronary microvasculature, many other techniques including cardiac magnetic resonance (CMR), computed tomography angiography (CTA), transthoracic Doppler echocardiography (TTDE), and myocardial contrast echocardiography (MCE), have emerged with great promise for the assessment of CMD (7–11). In the past 2 decades, MCE, a bedside, low cost, radiation-free technique, has gained growing evidence and exhibited promising diagnostic performances that were comparable to PET for determination of microvascular dysfunction in a variety of cardiovascular diseases (12). This review aims at discussing CMD lying in different clinical settings with the use of MCE. We will also briefly discuss the sonothrombolysis of contrast ultrasound and the incorporation of artificial intelligence (AI) into echocardiography.

## Normal Physiology of Coronary Blood Perfusion

**Figure 1:**
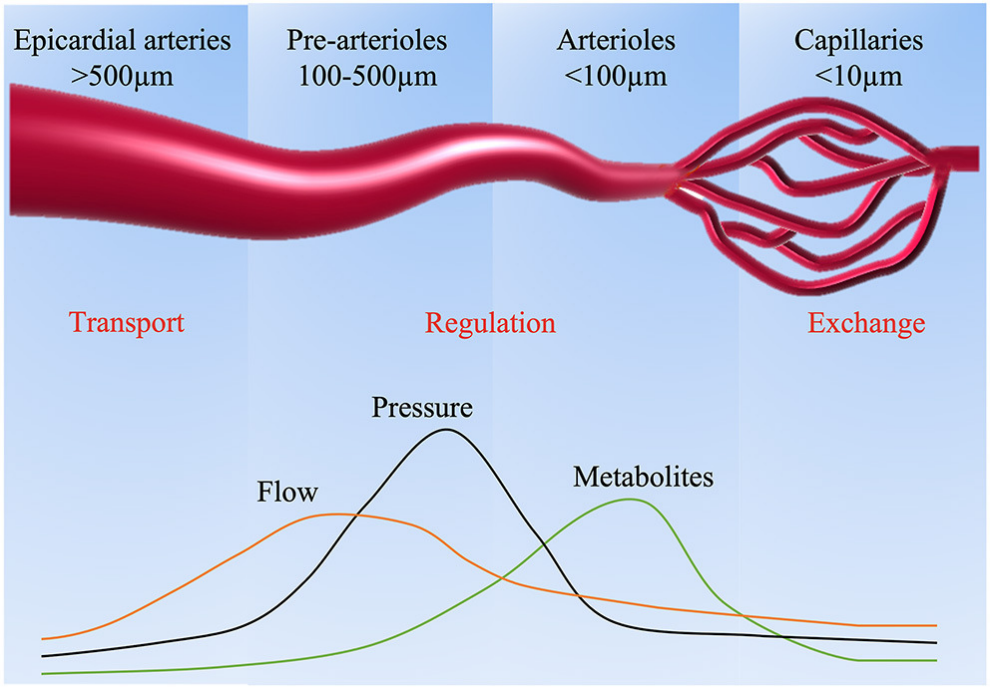
Coronary arterial circulation and mechanisms inducing vasodilation.

Myocardial perfusion is mainly governed by dynamic and combined changes in the epicardial coronary vasculature and microcirculation. Under resting conditions, the myocardium extracts roughly 75% of the blood oxygen with minimal reserve for additional oxygen extraction (13). Thus, the regulation of myocardial blood flow (MBF) becomes especially critical to keep balance between oxygen consumption and demand, which mainly resides in the microcirculation of coronary vasculature. Based on the size of the arterial structure, the capacitance and the resistance to MBF, the coronary arterial vasculature is divided into three compartments (Figure 1). The large epicardial coronary arteries (5 mm to 500 μm in diameter) have a capacitance function and normally offer negligible coronary resistance to blood flow. Epicardial coronary arteries can increase their blood content by around 25% and accumulate elastic energy by dilating during systole. The elastic energy is then converted into blood kinetic energy at the beginning of diastole. The pre-arterioles vessels (100–500 μm in diameter) represent the middle compartment, with a measurable drop in pressure. Their main function is to maintain pressure within a narrow range at the origin of the arterioles in response to changes in coronary perfusion pressure or flow. Finally, the distal compartment is made of arterioles (diameter <100 μm), which have the largest pressure drop compared to other compartments, characterized by matching blood supply to myocardial oxygen consumption with the tone influenced by metabolites produced during myocardial metabolism.

Under normal conditions, MBF remains relatively stable across a large range of perfusion pressures as a result of autoregulation, which is achieved by dynamic regulation in the pre-arteriolar and arteriolar microcirculation through flow-mediated dilation and pressure-dependent intrinsic mechanisms (14). For instance, in response to metabolic stimulation, the arterioles dilate correspondingly, resulting in the decrease of resistance in the entire arteriolar network. Subsequently, the pressure in the pre-arterioles also reduces accordingly, causing dilation in the pre-arterioles and the large epicardial coronary (15). However, in the setting of CMD, the disruption of these adaptive mechanisms determines alterations in the blood supply, which can be assessed by various noninvasive methods.

## Coronary Microvascular Dysfunction

The term CMD, referring to the structural and functional abnormalities of coronary microvasculature, has gradually been recognized as an additional mechanism of myocardial ischemia in a variety of cardiovascular diseases with elevated risk for adverse outcomes (16). Owing to the growing knowledge of the pathophysiological mechanisms of CMD, this universal phenomenon regarding myocardial ischemia is not only confined to atherosclerosis of coronary microvasculature, but also presents in other clinical conditions including non-ischemic cardiomyopathies, takotsubo syndrome and HFpEF. From the clinical perspective, CMD can be assigned into 4 groups with several pathogenetic mechanisms, which may coexist in the same condition and their importance varies in various clinical settings (Table 1). Any pathogenetic mechanism that may disrupt the coronary microvasculature is responsible for CMD, encompassing coronary spasm, endothelial dysfunction, smooth muscle dysfunction, microvascular inflammation, microvascular rarefaction, and extramural compression (17). These structural and functional abnormalities determine alterations in the coronary blood supply, leading to a blunted MBF and ultimately resulting in myocardial ischemia. Structural abnormalities associated with CMD are mainly manifested as luminal narrowing of the intramural arterioles and capillaries, capillary rarefaction, perivascular fibrosis and microvascular remodeling, often presenting in patients with hypertensive heart disease and hypertrophic cardiomyopathy (HCM) (18). As a consequence of medial wall thickening and different degrees of intimal thickening by smooth cell hypertrophy and increased deposition of collagen in these clinical conditions, coronary physiology and coronary blood flow (CFR) were subsequently impaired. Functional alterations are mainly associated with the presence of an impaired dilatation or an increased constriction of the coronary microvessels (19). The role of impaired microvascular vasodilator capacity is characterized as suboptimal coronary vasodilator response to exercise or pharmacological stress. In addition, increased vasoconstriction can be detected by intracoronary provocative testing with acetylcholine. It should be noted that the functional responsiveness of coronary microcirculation can be affected by some factors, including blood pressure, heart rate and diastolic time.

**Table 1:** Classification of coronary microvascular dysfunction.

	**Clinical settings**	**Pathogenetic mechanisms**
CMD in the absence of obstructive CAD and myocardial diseases	Microvascular angina	Endothelial dysfunction
	Risk factors for cardiovascular diseases
Smooth muscle dysfunction	HFpEF	Microvascular remodeling
Microvascular remodeling	Hypertrophic cardiomyopathy	Smooth muscle dysfunction
CMD in the presence of myocardial diseases without obstructive CAD	Takotsubo cardiomyopathy	Vascular rarefaction
	Dilated cardiomyopathy	
	Diabetic cardiomyopathy	
	Aortic stenosis	
	Amyloidosis	
Vascular wall infiltration	Atherosclerotic acute coronary syndrome	Endothelial dysfunction Smooth muscle dysfunction
Luminal obstruction Extramural compression	Stable CAD	
CMD in the presence of obstructive CAD		
Luminal obstruction (microembolization)	PCI	Luminal obstruction
CMD related to interventional procedures	Coronary artery grafting	Autonomic dysfunction

*CAD, coronary artery disease; CMD, coronary microvascular dysfunction; HFpEF, heart failure with preserved ejection fraction; PCI, percutaneous coronary intervention*.

Although the coronary microvasculature cannot be directly visualized *in vivo* due to the restriction of current techniques, CMD can be functionally expressed as reduced CFR by several invasive and non-invasive techniques, which is the ratio of the maximum increase in coronary flow to the resting value after pharmacological vasodilatation and represents an integrated measure of epicardial stenosis severity and microcirculation. In the absence of coronary epicardial artery stenosis, reduced CFR is a marker of CMD. Reduced CFR has been described in a certain number of patients with chronic coronary syndromes and those with acute coronary syndromes (ACS) in the absence of coronary epicardial obstruction, and is also associated with a higher risk of major adverse cardiovascular events, including myocardial infarction, progressive heart failure, and sudden death (20, 21). Traditionally, CFR has referred to the invasive measurement of flow reserve, while myocardial perfusion reserve (MPR) is used in this review to focus on the non-invasive measurement methods. Other potential imaging parameters have also been demonstrated to be associated with CMD, including MBF velocity, myocardial blood volume (MBV) and coronary flow velocity reserve (CFVR) (22, 23).

## Current Status of CMD Assessment by Non-invasive Imaging Modalities

**Figure 2:**
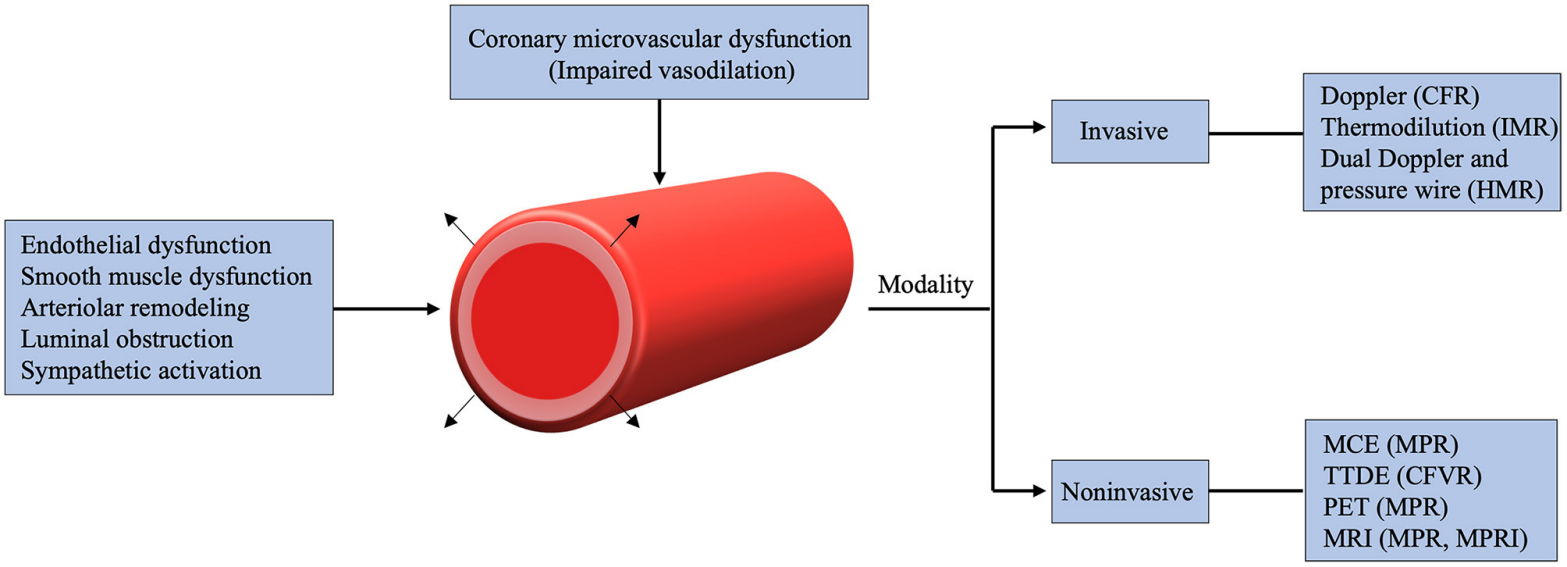
Mechanisms and various imaging modalities of coronary microvascular dysfunction. CFR, coronary flow reserve; CFVR, coronary flow velocity reserve; HMR, hyperemic microvascular resistance; IMR, index of microcirculatory resistance; MCE, myocardial contrast echocardiography; MPR, myocardial perfusion reserve; MPRI, myocardial perfusion reserve index; TTDE, Transthoracic Doppler echocardiography; MRI, magnetic resonance imaging; PET, positron emission tomography.

CMD can be assessed by several invasive and non-invasive modalities (Figure 2). More recently, almost all the non-invasive cardiac imaging modalities have been used to explore the mystery of CMD. Characterizing CMD requires reliable and reproducible measurements which allow for better understanding of its pathophysiological mechanisms. Notably, unlike invasive method, non-invasive techniques only assess the vasodilator capacity of vascular smooth muscle cell, focusing on the identification of impaired vasodilatory capacity of the coronary microcirculation. Along with the advances in current techniques, non-invasive assessment of CMD can now be obtained by several imaging techniques, including PET, CMR, CTA, TTDE and MCE (Table 2).

**Table 2:** Various invasive and non-invasive imaging modality and their advantages and disadvantages.

**Modality**	**Principle**	**Assessment of CMD**	**Contrast media**	**Advantages**	**Disadvantages**
Myocardial Contrast echocardiography	Continuous infusion of gas-filled microbubbles until reaching a stable concentration in the blood, followed by thoroughly destruction of microbubbles	The concentration in the myocardium provides the cross-section area, whereas the rate of reappearance of microbubbles gives the mean velocity. MBF can be quantified by multiplying these 2 variables	Microbubbles (Optison, Definity, SonoVue)	• Bedside • Radiation-free • Wide availability • Minimal risk • Inexpensive	• Operator-dependent • Suboptimal images in some situations (obesity, lung disease) • Without FDA approved for perfusion • Fewer validation studies compared to other modalities
Transthoracic Doppler echocardiography	Pulsed-wave Doppler sampling of the proximal left anterior descending coronary artery	CFVR is the ratio of coronary flow velocity at stress and rest	Microbubbles (Optison, Definity, SonoVue)	• Bedside • Radiation-free • High feasibility • Correlated well with intracoronary Doppler wire	• Operator-dependent • Suboptimal images in some situations (obesity, lung disease) • Poor correlation with PET • Limited data in nonobstructive CAD
PET	Dynamic first-pass vasodilator stress and then rest perfusion imaging	Post-processing software that performs automated segmentation and AIF measurements during dynamic first pass scanning	PET tracers (^15^O-labeled water, ^13^N-labeled ammonia ^18^F-flurpiridaz)	• Most validated modality • Good reproducibility • Extensive prognostic data • Can assess segmented MBF	• Radiation exposure • Expensive • Not widely available
CMR	Dynamic first-pass vasodilator stress and then rest perfusion imaging	Exploits the first-pass kinetics of T1-enhancing extracellular gadolinium-based contrast media and the increase in signal intensity is proportional to the perfusion and blood volume	Gadolinium-based	• No radiation exposure • Excellent spatial resolution • Provide tissue characterization in the same study • Validated against invasive measurements	• Expensive • Not widely available • Few prognostic data • Difficult for patients (frequent breath holds) • Time-consuming • Limited in renal failure
CT	Dynamic first-pass vasodilator stress and then rest perfusion imaging	Permits serial myocardial and left ventricular cavity sampling for quantifying blood flow, based on first-pass detection of the maximum slope of the time-attenuation curve in the target tissue, divided by the maximum arterial input function	Nonionic iodine	• Anatomic coronary data and perfusion data in one same study	• Radiation exposure • Risk for contrast-induced nephropathy • Limited validation in non-obstructive CAD • Iodinated contrast can cause vasodilation leading to overestimation of MBF
Intracoronary coronary flow reserve	Coronary flow reserve, assessed as coronary flow velocity reserve	Use an intracoronary Doppler flow wire or a temperature sensor-tipped guidewire to measure the ratio of maximal hyperemic to resting coronary blood flow	Nonionic iodine	• Gold standard • Most common method	• Invasive • Expensive
Index of microcirculatory resistance	Index of microcirculatory resistance	Use a pressure/temperature wire to measure the ratio of distal coronary pressure/thermodilution-derived mean transit time during maximal hyperemia	Nonionic iodine	• Independent of epicardial vascular function • Reproducible	• Requires a specialized wire • Variability may result from the injection of intracoronary saline bolus
Acetylcholine testing	Changes in coronary blood flow and coronary microvascular spasm	Intracoronary acetylcholine infusion with ECG monitoring.	Nonionic iodine	• Allow assessment of coronary constrictive properties	• Limited data • Adverse effects

*AIF, arterial input function; CAD, coronary artery disease; CFVR, coronary flow velocity reserve; CMD, coronary microvascular dysfunction; CMR, cardiac magnetic resonance; CT, computed tomography; FDA, U.S. Food and Drug Administration; MBF, myocardial blood flow; PET, positron emission tomography*.

### Nuclear Imaging

PET is the mostly used and well-validated non-invasive technique for the quantification of MBF and is now considered the gold standard for non-invasive assessment of CMD. It involves the utility of post-processing software that performs automated segmentation and arterial input function measurements during dynamic first pass scanning to assess the regional and global rest and stress MBF (24). PET derived MPR showed a good correlation with adverse prognosis in patients with coronary syndromes in several prospective studies (25, 26). The predictive value of reduced MPR for cardiac events has also been described in different subsets of populations, such as in women (27), HCM (28), cardiometabolic disease (29), diabetes mellitus (30), and chronic kidney disease (31). Based on these strong data, MBF and MPR by PET may allow for the risk stratification in some clinical conditions. Furthermore, these parameters have been used as a surrogate marker for coronary vascular health and to monitor therapeutic interventions (4, 32, 33), and this image-guided cardiovascular therapy may have great significance for clinical decision making in the future. Despite well-validated diagnostic and prognostic data by PET, its use is restricted in clinical practice due to some limitations including high radiation exposure, time consuming, limited availability and high cost.

### CMR

The utility of MBF and MPR quantification by CMR allows a simultaneous assessment of coronary anatomy, functionality and myocardial perfusion. It exploits the first-pass kinetics of T1-enhancing extracellular gadolinium-based contrast media and the increase in signal intensity is proportional to the perfusion and blood volume as the contrast medium diffuses into the interstitial space from the microvasculature (34). There was a good agreement in global MBF measurements between CMR and PET (*r* = 0.92, *p* < 0.001) in patients with stable CAD (35). The predictive value of MBF obtained by stress perfusion CMR for adverse cardiac events has also been validated in specific risk populations such as in patients with cardiovascular risk factors (36), non-obstructive CAD (37), acute myocardial infarction (AMI) after revascularization (38) and HCM (39). Nevertheless, several shortcomings of this technique should be noted, including time-consuming for scan and post-processing, interobserver variability, lack of widespread availability and imaging artifacts. More studies are needed to demonstrate the clinical utility of assessment of CMD by CMR.

### Computed Tomography

CTA in combination with CT perfusion (CTP) has the ability to obtain coronary anatomic and myocardial perfusion in one examination (40). Dynamic CTP permits serial myocardial and left ventricular cavity sampling for quantifying blood flow, based on first-pass detection of the maximum slope of the time-attenuation curve in the target tissue, divided by the maximum arterial input function. It provides good diagnostic accuracy for the detection of perfusion defects compared with CMR (41). However, CTA does not have any advantages over other imaging techniques, and its application to assess CMD is restricted in regular clinical practice currently. Further functional information regarding the hemodynamic evaluation of lesion specific ischemia can be obtained by CTA-derived fractional flow reserve (FFR), which involves a 3-dimensional derived coronary model to stimulates maximal hyperemia and quantify MBF (42). It seems to be a promising tool to assess microvascular function in patients with CTA defined intermediate stenosis (43). Despite the potential for identification of CMD, major limitations of this technique include the high effective radiation dose, beam hardening artifacts and restricted use of iodinated contrast agents in renal insufficiency.

### TTDE

CFVR by TTDE, is the ratio of coronary flow velocity at stress and rest obtained by the pulsed-wave Doppler sampling of the left anterior descending (LAD) coronary artery in the absence of epicardial artery stenoses (44). CFVR correlates well with flow acquired by an intracoronary Doppler wire (45) and cutoff values ≤ 2–2.5 are commonly used as indicative for impaired coronary microvascular function. CFVR reveals its diagnostic and prognostic capacity in various populations such as in CAD (46, 47), ACS (48), diabetes (49), and HFpEF (50). Advantages of TTDE are low cost, radiation-free, high feasibility, but it is operator dependent, and the image quality affected by artifacts in obese or patients with lung disease needs to be taken into account.

## Basic Principles of Myocardial Contrast Echocardiography (MCE) for Assessment of CMD

### Ultrasound Contrast Agents

MCE is a non-invasive, bedside and inexpensive technique developed in the past 3 decades, exploiting strong backscatter and non-linear behavior of ultrasound enhancing agents (UEA) to help improve endocardial border definition, detect myocardial perfusion abnormalities and quantify MBF (51). Contrast echocardiography microbubbles are UEA that consist of a hemodynamically inert gaseous core and a stabilizing outer shell, which oscillate under the influence of ultrasound waves. These microbubbles containing the low diffusible and low solubility gas have tiny size (smaller than 10 μm) and similar rheology behavior as red blood cell, which can traverse the smallest human blood vessels, the capillaries, without disrupting the local environment, resulting in opacification of the left ventricle and myocardium. Currently, the most used contrast agents are Sonovue (Bracco, Milan, Italy), Optison (Amersham Health AS, Oslo, Norway), and Definity (Bristol-Myers Squibb, Billerica, Massachusetts). There are some differences in the shell compositions and gas cores among these agents, but all are suitable for MCE. Furthermore, these agents have been evaluated extensively, showing both safe and effective in patients with various cardiovascular diseases as well as in pediatric patients.

### Physical Principles

It was discovered that microbubbles were gradually destroyed at a high mechanical index (MI > 0.3) as they transited through the myocardial microcirculation. Notably, with the non-linear oscillation effect of microbubbles under low MI ultrasound, different reflections can appear compared with tissue, providing a better differentiation by imaging techniques. Based on these characteristics of microbubbles, by either triggering ultrasound to one frame every cardiac cycle or by using a very low mechanical index (VLMI) (<0.2) imaging, myocardial contrast enhancement can be visualized by intravenous injections (52). A landmark discovery was made that the triggering technique could be used to quantify MBF, and therefore it provides important bedside information on MBF during stress echocardiography in various clinical conditions (11). Initially, high MI-triggered imaging was used to analyze myocardial perfusion (53), but it has gradually been replaced by VLMI imaging which permits simultaneous assessment of wall motion and myocardial perfusion (54). When a high MI is used, the ultrasound beam destroys the microbubbles, resulting in nearly complete bubble destruction with every pulse. Triggering ultrasound to one frame timed to end systole in the cardiac cycle at a sequence of incrementally longer cardiac cycles allows a replenishment of contrast agent corresponding to flow to the given region during that time sequence. With longer triggering intervals, more microbubbles replenish the capillaries and higher signal intensity appears until finally a plateau phase is reached. Thus, when imaging at a low MI in real time, brief high MI impulses can be used to the imaging plane, after which replenishment can be visualized in real time at the low MI. Calculation of MBF depends on the microvascular cross-sectional area as well as mean velocity of microbubbles during a constant venous infusion of microbubble contrast. When reaching a stable concentration of microbubbles in the blood during continuous infusion, high MI ultrasound is utilized to thoroughly destroy microbubbles and the rate of reappearance of microbubbles gives the mean velocity, whereas the concentration in the myocardium provides the cross-section area (55). Thus, MBF can be quantified by multiplying these 2 variables together. Intensity values “VI” fitted to a monoexponential function: VI = A × (1-e^−β*t*^) where VI is the video intensity at pulse interval t, A is the plateau video intensity reflecting the microvascular cross-sectional area and β gives the rate constant that determines the rise of video intensity after bubble destruction representing the velocity of the microbubbles. The product of A and β represents MBF and has shown an excellent relationship with the radiolabeled microsphere-derived MBF in experimental animals (*r* = 0.96, *p* < 0.001) (11). When combined with stress technology, MPR can be obtained as well as MBF velocity reserve and MBV reserve. Due to attenuation or some technical reasons, the inhomogeneous contrast enhancement of the myocardium can be adjusted using ratio of myocardial video intensity to the adjacent left ventricular cavity. Different ultrasound contrast imaging techniques, each with relative advantages and pitfalls, are currently applied to utilize the specific reflection features for microbubbles vs. tissue, including pulse inversion, power modulation and coherent contrast imaging. These techniques are multi-pulse schemes that can cancel linear reflections from tissue and enhance non-linear reflections from microbubbles, which allows for high sensitivity contrast imaging with less signal-to-noise ratio.

### Qualitative and Quantitative MCE

CMD is diagnosed when there is inadequate visual microvascular refill within 2 s after the destruction of high MI (56). MCE can detect microvascular perfusion abnormalities in 46.8% (range, 41–52%) of patients with no obstructive CAD, which is similar to that obtained invasively in the WISE registry (57). However, visual qualitative MCE fails to detect abnormal FFR in every vessel manifesting abnormal microvascular perfusion (58). With various quantitative parameters, quantitative MCE has the potential to identify pathologic microvascular patterns in patients with CMD. A significantly lower hyperemic MBF and a lower β reserve were found in patients with CMD (56). This phenomenon can also be found in patients with cardiac syndrome X or diabetes by MCE (44, 59). In addition, MCE derived MBF reserve and β reserve were highly correlated with CFVR by TTDE in patients with cardiac syndrome X (44). A recent study also showed that an MBF reserve <2 by MCE classified 37% of patients with chest pain but no obstructive CAD as having CMD (60). Although there is no consensus regarding the diagnostic criteria, data regarding CMD by MCE is growing as the rapid development of MCE. There is an increasing interest in the role of MCE in the evaluation of CMD involved in various kinds of diseases.

## Assessment of CMD by MCE in Different Disease Categories

### Myocardial Ischemia Without Obstructive CAD

The past paradigm of ischemic heart disease was the well-established link between myocardial ischemia and obstructive atherosclerosis of epicardial coronary arteries. Coronary angiography has been recognized as the gold standard to evaluate the severity and extent of CAD. Nevertheless, growing evidence indicates that angiography frequently fails to detect obstructive CAD in patients with angina symptoms suggestive of myocardial ischemia in clinical practice (61). Thus, this paradigm has been challenged by increasing clinical data, which has led to the gradual recognition that CMD is one of the mechanisms responsible for myocardial ischemia and symptoms in ischemia with non-obstructive coronary artery disease (INOCA) and constitute the clinical picture of primary microvascular angina (MVA). Importantly, contrary to the past understanding, there is a worse prognosis, a poor physical functioning and a reduced quality of life in patients with INOCA based on the data from the National Heart, Lung, and Blood Institute-sponsored WISE (Women's Ischemia Syndrome Evaluation) (16).

On the basis of ischemic cascade, where abnormal perfusion precedes wall motion abnormalities during increased demand induced ischemia, a large number of MCE studies have demonstrated that perfusion analysis provides an incremental benefit for CAD detection over wall motion abnormalities alone in the setting of stress echocardiography (62–64). However, it has been frequently encountered in clinical practice that an inducible defect within a given coronary artery territory does not have a significant obstructive lesion detected at angiography. In a MCE study involving 380 consecutive patients referred for coronary angiography, 91 patients had abnormal myocardial perfusion at peak stress but without a significant epicardial stenosis, implying the potential role of CMD with myocardial ischemia in patients with chest pain. More importantly, these patients appeared to have a 2-fold higher likelihood of adverse events at 4-year follow-up compared with those with negative myocardial perfusion and normal or non-obstructive angiography, indicating that patients with false-positive stress perfusion studies should be followed closely and evaluated for new therapeutic strategies (65). Apart from the qualitative analysis of myocardial perfusion, non-invasive quantification of MPR can also be assessed by MCE, showing a good correlation with Doppler flow wire (*r* = 0.76, *p* < 0.001) (66). For the majority of patients with CAD (<85% luminal diameter narrowing of the coronary arteries), resting MBF is normal or even higher as <300-μm arterioles distal to it dilate in order to maintain myocardial oxygen supply. Interestingly, this phenomenon was also presented in patients with INOCA. Since microvessels in these regions have already used some of their reserve to maintain normal resting MBF, MBF cannot increase during stress to the same extent as in other regions with greater microvascular reserve. These findings were further elucidated where the MCE derived MPR was significantly blunted in 18 women with INOCA compared with controls, implying the coronary resistance vessels as the site of microvascular dysfunction (67).

### Obstructive CAD: Atherosclerotic Acute Coronary Syndrome

**Figure 3:**
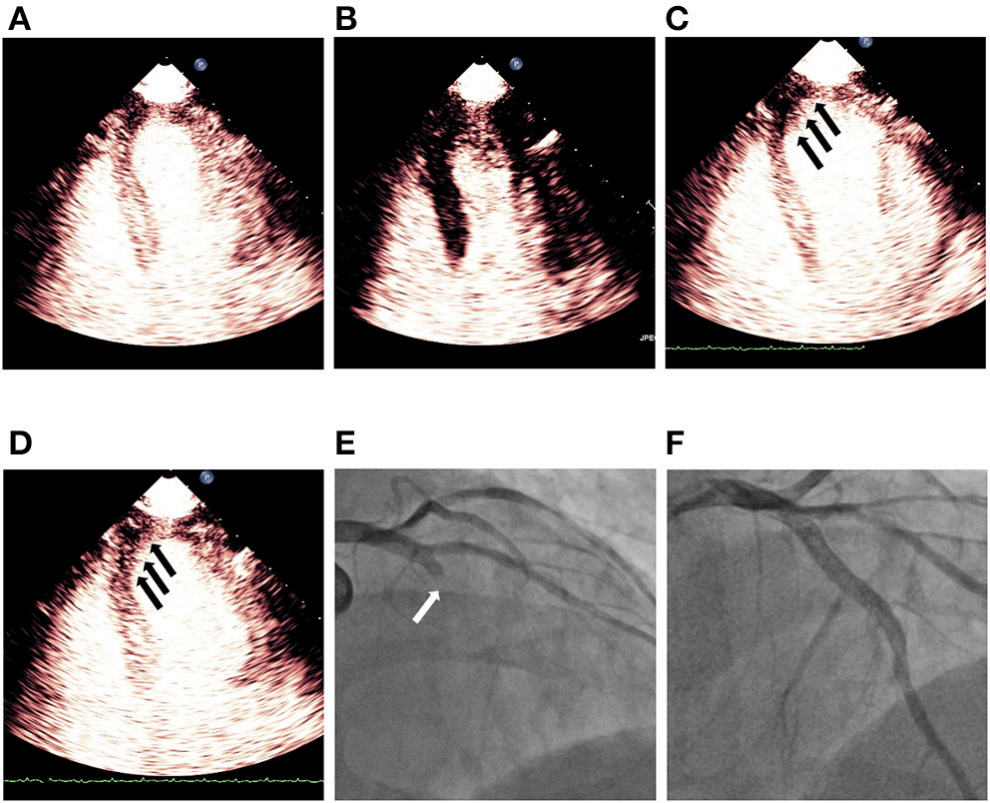
Demonstration of persistent no reflow phenomenon noticed on MCE after successful revascularization of LAD STEMI. **(A)** Shows myocardial contrast replenishment before the high MI impulse, while **(B)** indicates complete microbubble destruction immediately after the high MI impulse. **(C)** Shows a defect (*black arrows*) in the segments of the infarct zone during replenishment (~4 beats post-high MI impulse), while **(D)** indicates a persistent defect (*black arrows*) in LAD territory although a plateau intensity (~10 beats after the high MI impulse) had been reached. **(E)** Shows blocked LAD (*white arrow*) before PCI and **(F)** indicates revascularization of LAD after successful PCI. LAD, left anterior descending; MCE, myocardial contrast echocardiography; PCI, percutaneous coronary intervention; STEMI, ST segment elevated myocardial infarction.

The “no reflow” phenomenon refers to the inability to reperfuse the coronary microcirculation in a previously ischemic region despite opening of the epicardial vessel and thrombolysis in myocardial infarction (TIMI) grade 3 flow on coronary angiography after percutaneous coronary intervention (PCI) in patients with AMI (68). This phenomenon was first described by Ito et al. (69) and subsequently confirmed by multiple MCE studies (70, 71). It is related to the structural and functional abnormalities of coronary microcirculation and extravascular compression. The microvascular obstruction and the damage of tissue and microvasculature during myocardial ischemia were believed to be the main causes of the no reflow phenomenon. Microvascular obstruction may result from distal atherothrombotic embolization as well as myocardial edema and inflammation leading to microvascular compression. The no reflow phenomenon seems to be associated with the duration of ischemia, determining the occurrence and extent of no reflow zone regardless of distal microembolization (72). Despite the wide range of interventional and pharmacologic therapeutic strategies for STEMI management, the no reflow phenomenon may still be present in a significant percentage of patients, especially in patients with myocardial infarction involving the LAD territory (Figure 3), with a poor prognosis including higher incidence of LV remodeling, heart failure (HF) and death. A MCE study showed that >60% of patients with STEMI after successful PCI still had some form of microvascular perfusion abnormality, and the occurrence of the no reflow phenomenon was found to be associated with poor recovery of LV systolic function at 6-month follow-up and was the most significant variable in predicting major adverse cardiac events over the 1-year follow-up period (68). Given the association of the no reflow phenomenon after reopened infarcted arteries with relatively poor clinical outcomes, it has become a treatment target for medical and pharmacological interventions and may be useful for risk stratification. Several studies demonstrated that pharmacological interventions and occlusive protection devices may increase microvascular perfusion or reduce the no reflow area, raising the possibility for individualized intervention which may be beneficial to cardiac outcomes by addressing the no reflow phenomenon (73, 74).

### Myocardial Diseases: HFpEF; Hypertrophic Cardiomyopathy; Takotsubo Cardiomyopathy

HFpEF is characterized by the signs and symptoms of HF despite a normal or near-normal EF, which has become a challenge for diagnosis and treatment in clinical practice. With common comorbidities such as hypertension, diabetes mellitus and metabolic syndrome in HFpEF, its pathophysiological mechanisms appear to be complicated and heterogeneous, including arterial stiffness, ventricular-arterial coupling, vascular endothelial cell inflammation and dysfunction, and chronotropic incompetence. The role of CMD has gained growing consent over years in the pathophysiology of HFpEF by an increasing number of studies (75). Recent advances in non-invasive imaging modalities enable accurate quantification of MPR, presenting a good correlation with that of invasive method in many cardiovascular diseases, including HFpEF. Prevalence and correlates of CMD in HFpEF patients were analyzed with echocardiography in the PROMIS-HFpEF study. Seventy-five percentage of HFpEF patients had CMD (defined as CFVR <2.5) in this prospective multi-center study and the degree of CMD was shown to correlate with markers of HF severity (76). Apart from the high prevalence of CMD in HFpEF, the impairment of MPR also directly correlated with excise capacity, which is an independent predictor of reduced exercise tolerance (77, 78). Furthermore, CMD was also found to correlate with markers of systemic endothelial dysfunction, suggesting HFpEF as a systemic disorder associated with endothelial dysfunction and microvascular disease (79). However, it remains to be determined whether CMD is the primary cause of ventricular remodeling and HFpEF, or the myocardial remodeling property of HFpEF may lead to CMD. A follow-up of PROMIS-HFpEF study showed a strong association between the presence of CMD and the risk of adverse cardiovascular outcomes including cardiac death and HF hospitalizations at follow-up, raising the awareness of the urgent need to gain more insight into the pathophysiology and therapeutic advances at improving prognosis in patients with HFpEF (50). Currently, the non-invasive assessment of CMD in HFpEF mainly resides in PET and CMR. However, with the capacity to clearly present endocardial border, MCE is particularly useful in determination of LVEF compared with traditional echocardiography, which is of great importance in the diagnosis in patients with HF and can provide prognostic information. Moreover, unlike other non-invasive techniques, MCE can assess LV function and MPR in a single examination, simultaneously providing structural and functional information for diagnosis and prognostic evaluation. Based on these, MCE may be helpful to address the pathophysiological mechanisms of CMD in patients with HFpEF, which needs to be investigated in future research.

HCM is characterized by unexplained left ventricular asymmetric hypertrophy and the absence of cardiac or systemic triggers. MCE has been proposed as a helpful diagnosis tool in questioned HCM and the precise determination of left ventricular wall thickness by MCE is of great significance in the prognostic assessment of patients with HCM (80). Recently, CMD, representing a predisposing factor for myocardial ischemia, has also been recognized as one of the most important pathophysiological features. The structural abnormalities, characterized by marked wall thickening of intramural coronary arterioles resulting in severe reduction in luminal area, are considered the most relevant substrate of CMD in the presence of increased oxygen demand. In addition to structural changes of small vessel, functional abnormalities have also been described, causing blunted MBF and MPR by several non-invasive imaging techniques (81, 82). The blunted MPR in HCM is mainly due to the failure of MBV to increase during hyperemia, as the affected segments have exhausted myocardial capillary autoregulation by presenting near-maximal capillary vasodilatation at rest. In agreement with previous studies in patients with HCM, these findings of myocardial microcirculation were also clearly shown in a MCE study, demonstrating a good correlation with PET (83). Noteworthy, CMD can be present not only in the hypertrophied myocardium, but also in the non-hypertrophied LV segments and the degree of microvascular dysfunction is related to the extent of LV hypertrophy (84, 85). The degree of CMD, expressed as the perfusion defect size by MCE, was found to correlate with decreased myocardial contractile property, indicating an important role of microvascular dysfunction in the process of HCM (86). Despite a generally benign prognosis in patients with HCM, a certain quantity of patients will progress to LV dysfunction and eventually heart failure, and sudden death owing to ventricular arrhythmia can be the first manifestation of the disease. The degree of CMD, by presenting severely blunted MBF following dipyridamole stress assessed by PET, was a strong, independent predictor of long-term clinical deterioration and death from cardiovascular causes in patients with HCM, suggesting CMD as a underlying target to address the adverse prognosis (87). Two invasive methods, alcohol septal ablation (ASA) and surgical myectomy are used to reduce LV outflow tract gradients in patients with obstructive HCM by relief of extravascular compression, which is also one of the causes of CMD. MCE is widely used to address ASA by precise identification of the coronary artery branch supplying the target septal zone. After relieving these extravascular compression forces with ASA, MCE-derived MPR improved, but did not normalize compared with healthy controls after 6 months (88). This could be partly explained by the residual vascular remolding and fibrosis that results in limiting myocardial perfusion in these patients. In recent years, a novel minimally invasive treatment, echocardiography-guided percutaneous intramyocardial septal radiofrequency ablation has shown promising results and the combination with MCE can be useful in the selection of ablation area and at follow-up in these patients (89).

Takotsubo cardiomyopathy (TC), also known as stress cardiomyopathy or apical ballooning syndrome, was identified in 1990 and is characterized by the sudden onset of chest symptoms, evidence of myocardial ischemia and transient left ventricular dysfunction without substantial angiographic stenosis. A number of pathophysiologic mechanisms have been proposed to explain the apical dysfunction observed in TC, including multivessel coronary vasospasm, spontaneous coronary thrombus lysis, abnormalities in coronary endothelial function and direct catecholaminergic effects on the myocardium (90). The involvement of CMD has also been advocated as a possible pathogenic mechanism underlying the wall motion abnormalities in TC by several invasive and non-invasive methods (91, 92). In the acute phase (1 day) of TC, reduced MBF and velocity were detected in dysfunctional LV segments compared with those with normal wall motion (93). These findings were also described in a prospective MCE study involving 11 patients (mean age, 70.9 ± 17.5 years; 8 women) received diagnoses of TC, suggestive of a potential pathophysiologic role of microvascular dysfunction (94). With uniquely favorable prognosis compared with other causes of ACS, it is often characterized by a rapid improvement of LV wall motion over a period of days or a few weeks. Nine consecutive patients with TC were serially followed by MCE at 1 day, within 1 week and 3–6 months after index admission (95). By 1 week, the relative improvement of LVEF was 26%, whereas myocardial perfusion had improved by nearly 50% and returned to normal by 3–6 months, suggesting a pronounced improvement in microvascular function preceding recovery of wall motion abnormalities. This transient and complete restoration of the myocardial microcirculation was less inclined to the fact that disruption of the microcirculatory architecture was caused by epicardial coronary artery occlusion or vasospasm. To further elucidate the underlying pathophysiological mechanism, Galiuto et al. found a clear perfusion defect within the dysfunctional myocardial area at MCE in 15 consecutive women (68 ± 14 years) with TC, which can be transiently reduced by adenosine and entirely resolved at 1 month follow-up, whereas no changes were observed in anterior STEMI patients (96). These findings were endorsed by other MCE studies, by exhibiting lower quantitative parameters (MBV, MBF and velocity) in the akinetic segments than in normokinetic segments in TC patients, and these parameters were further deteriorated in dysfunctional segments in patients with STEMI (96–98). Thus, the authors speculated that coronary microvascular constriction appears to be a potential mechanism as adenosine vasodilates constricted microvessels. The greater prevalence of TC among post-menopausal women, along with the association to physical or emotional stress, may support the hypothesis of stress-mediated vasoconstriction enhanced by estrogens depletion. However, it remains debated whether CMD is the primary cause, or a secondary phenomenon and further studies are needed to gain insights into the causes, triggers and mechanisms responsible for TC. Of importance, it has been reported that the degree of CMD, by presenting with coronary slow flow on coronary angiography, may have a worse clinical presentation and carry a poor prognosis (99).

## Applications of MCE in the Treatment of CMD

Contrast ultrasound has a variety of applications not only in diagnosing and providing prognostic information of cardiovascular disease, but also in the treatment of patients with STEMI through sonoperfusion and sonothrombolysis. When exposed to lower MI, the microbubbles expand and compress steadily (stable cavitation), whereas microbubbles will have greater volumetric changes and eventually collapse at higher MI, as known inertial cavitation (100). Multiple animal studies have shown that high MI impulses of diagnostic ultrasound can successfully dissolve acute intravascular thrombi mainly due to the effects of inertial cavitation, which causes increased shear stress and powerful jetting inducing direct mechanical destruction of the clot (101, 102). Noteworthy, even in the absence of upstream recanalization, diagnostic high MI impulses was demonstrated to have a positive effect on microvascular flow (103). This phenomenon can be partly explained by the role of cavitation-mediated adenosine triphosphate (ATP) release, which can be converted to adenosine for reducing vascular tone and increase nitric oxide (NO) synthesis, finally leading to vasodilatation (104). The first prospective randomized human study by MRUSMI group (Microvascular Reperfusion Utilizing Sonothrombolysis in acute Myocardial Infarction) used short pulse duration high MI pulses to examine the effects in 100 patients with STEMI, with each 50 of whom randomized to pre- and post-PCI sonothrombolysis and to PCI only, respectively (105). Patients in sonothrombolysis group had a higher recanalization rate and TIMI flow before PCI, and reduced infarct size on CMR, resulting in sustained improvements in LVEF and less need for intracardiac defibrillator placement at 6 months follow-up. Furthermore, a substudy showed improved LV global longitudinal strain and decreased perfusion defect size by MCE at follow up, and the only independent predictor of LV remodeling was treatment with sonothrombolysis (106). As contrast ultrasound is portable, this technique was also shown to have a promising feasibility of in-ambulance sonothrombolysis for STEMI (107). These findings have provided more insight into the background of sonothrombolysis by contrast ultrasound and additional multi-center prospective studies are needed to test the clinical effectiveness of this method. It is also very interesting to determine whether the sonothrombolysis will have a positive effect on patients with other cardiovascular diseases involving CMD as it can augment myocardial flow through mechanical and biological mechanisms.

Apart from enhancement of vascular compartments, microbubbles can be designed to carry drugs or genes and used to deliver site-specific therapy to targeted organs in the body. When activated by external ultrasound energy, the acoustic microspheres can serve as catalysts and vehicles of drug or gene packages, providing a direct and transient access to tissues and organs. Targets for molecular imaging, therapy and drug delivery with ultrasound have been described in several clinical conditions, such as in myocardial ischemia, vulnerable plague, vascular remodeling and inflammation. Of note, low dose fibrinolytic therapy combined with targeted microbubbles appears to enhance sonothrombolysis efficacy by localizing and binding cavitation nuclei directly to the clot, which may be beneficial in the settings where primary PCI is not available emergently (103). Moreover, induced by contrast ultrasound, the increased permeability of cells and vessels can be used to delivery genetic material. There have been preclinical trials showing improved blood flow with targeted gene delivery microbubbles in animal models with vascular disease, implying a new interest in treatment strategies for these conditions (108). Nevertheless, it remains challenging for combined microbubbles to be fully incorporated into the clinical practice.

## Potential Applications of AI in MCE

Artificial intelligence (AI) has been incorporated into diverse areas of clinical medicine, especially in cardiovascular imaging analysis, which can address observer variability during imaging acquisition and interpretation. Recently, using AI quantification of CMR perfusion mapping involving over 1,000 patients with known or suspected CAD, both reduced MBF and MPR were associated with death and major adverse cardiovascular events independently of other clinical risk markers (109). AI is becoming a hotspot particularly in echocardiography compared to other techniques, which is more affected by interobserver variability and is strongly dependent on the expertise of operators. The applications of AI in echocardiography has shown promising results in view classification, automated analysis of size and function, wall motion abnormality, diagnosis of cardiovascular diseases and event prediction. In the field of MCE, despite its promising diagnostic performance in various clinical conditions, it is limited by time-consuming manual segmentation and requirement of high level of expertise and training. Of note, there have been several studies demonstrating that AI-empowered MCE allows a fully automatic approach for fast and accurate segmentation of myocardium, overcoming the above limitations by exploit different machine learning techniques (110). However, the current AI on MCE is still in its infancy, but it stands a chance of being utilized in routine clinical practice, improving the accuracy of screening of early cardiovascular diseases, and achieving early detection, intervention and treatment for a better prognosis.

## Conclusion

CMD is prevalent across a broad spectrum of cardiovascular diseases with a significant impact on prognosis. Current research provides good evidence that MCE is capable of assessing myocardial perfusion and quantifying MBF, addressing and advancing the pathophysiologic role of CMD in heterogeneous clinical conditions. Apart from the promising performance for diagnosis and prognosis in various types of cardiovascular diseases, contrast enhanced ultrasound also reveals therapeutic capacity to dissolve clot and restore microvascular function in the setting of STEMI. With the advent of targeted microbubbles, microbubbles attached drugs or genes may be used to deliver site-specific therapy to targeted organs. Finally, MCE combined with AI possesses a promising application prospect, and may be a feasible method to be used in the future. However, there is still limited knowledge about CMD in all of these conditions, indicating that additional research in this area is warranted to fully implement MCE into the daily workflow in clinical practice.

## Author Contributions

All authors participated in writing and critically reviewing this manuscript. All authors have read and agreed to the published version of the manuscript.

## Funding

This study was funded by Guangdong Natural Science Funds for Distinguished Young Scholar (Grant 2016A030306028), Guangzhou Science and Technology Program (Grant 201506010021), and Foundation of President of Nanfang Hospital (Grant 2020Z006 and 2018Z018).

## Conflict of Interest

The authors declare that the research was conducted in the absence of any commercial or financial relationships that could be construed as a potential conflict of interest.

## Publisher's Note

All claims expressed in this article are solely those of the authors and do not necessarily represent those of their affiliated organizations, or those of the publisher, the editors and the reviewers. Any product that may be evaluated in this article, or claim that may be made by its manufacturer, is not guaranteed or endorsed by the publisher.
